# 

**Published:** 1868-07-01

**Authors:** 


*CONTENTS:*

ORIGINAL PAPERS:

Progressive Paralysis of the Insane. By A. W. Bosworth, M. A., Paris, France.......... 411

Experimental Medicine. By Walter Hay, M.D., Chicago.......... 417

Case of Self-Mutilation. By Curtis T. Fenn, M.D., Chicago........ 429

Case of fracture of the Vertebra. By R. G. Bogue, M.D., Chicago... 431

Rush Medical College—Spring Course of Lectures............... 433

CORRESPONDENCE.......... 435

EDITORIAL.......... 440

ITEMS.......... 442

